# Comparison of preoperative trans-thoracic echocardiography with intraoperative findings in patients with congenital heart disease undergoing surgery: a prospective observational study

**DOI:** 10.1186/s13019-021-01711-8

**Published:** 2021-11-13

**Authors:** Josiah Miner Njem, Frank Edwin, Mark Tettey

**Affiliations:** 1 National Cardiothoracic Centre, Accra, Ghana; 2 University of Ghana School of Medicine and Dentistry, Accra, Ghana; 3 Department of Surgery, Jos University Teaching Hospital, PMB 2076, Jos, Plateau State Nigeria

**Keywords:** Trans-thoracic echocardiography, Congenital heart disease, Intra-operative findings

## Abstract

**Objective:**

To determine the diagnostic accuracy and safety of trans-thoracic echocardiography alone for indicating surgery by correlating preoperative trans-thoracic echocardiography with intra-operative findings in patients with congenital heart disease (CHD) in a low resource, low volume center.

**Methodology:**

The pre-operative trans-thoracic echocardiography and intra-operative findings of two hundred and fifty patients with CHD, undergoing surgery at the National Cardiothoracic Centre (NCTC), Korle Bu Teaching Hospital, from 2012 to 2017 were prospectively compared. Included in this prospective study, were all patients with CHD who had trans-thoracic echocardiography alone at the NCTC. Excluded were patients who were operated at the NCTC based on echocardiography done elsewhere, those who had echocardiography at the NCTC but were operated elsewhere, as well as those whose operative decision were based on cardiac catheterization or CT angiography and patients with acquired heart defects. The analysis included profiling of patients on different demographic and clinical parameters. SPSS software was used for analysis.

**Results:**

Of the 250 patients ages ranged from 2 months to 60 years. The mean was 4 years 95 days, median 1 year 180 days. The female sex accounted for 152 (60.6%). The preoperative trans-thoracic echocardiography correlated with intra-operative findings completely in 228 (91.2%) of patients, affirming the accuracy of this imaging modality. There were however, 19 (7.6%) false negatives and 3 (1.2%) false positive. Neither the false positive nor false negative errors resulted in complications or adversely affected the surgical outcome.

**Conclusion:**

Based on the results of this study, preoperative transthoracic echocardiography done by cardiologists at the National Cardiothoracic Center, Korle Bu Teaching Hospital Accra, demonstrated a high correlation with intraoperative findings. Echocardiography also proved to be sensitive, accurate and safe for indicating surgery in patients with congenital heart disease.

## Background

Transthoracic echocardiography has become increasingly popular in clinical practice [1–10]. It is used for the functional and anatomic evaluation of patients with various cardiac defects. Echocardiography enables direct visualization of the various chambers of the heart, intracardiac structures as well as proximal parts of major connecting vessel such as the aorta and pulmonary artery. This imaging modality is often used by most institutions as a primary non-invasive diagnostic investigation for the diagnosis of congenital cardiac defects, cardiac catheterization being indicated only in patients with complex congenital cardiac anomalies, when aorta and pulmonary artery anatomy cannot be clearly defined as well as in abnormal coronary artery origin or course [2–6, 10–16].

Although echocardiography is well established as the first line imaging technique for the diagnosis of all forms of congenital heart disease (CHD), most institutions continue to perform invasive imaging modalities such as cardiac catheterization prior to complete repair of these heart defects [1, 2, 6–20]. Its main advantage is that it is non-invasive, easy to perform, reproducible and cost effective. Its main draw-back is that it is operator dependent [1–8, 17–21].

In the developing nations such as the West African sub-region where most institutions lack facilities, echocardiography is therefore the mainstay for the diagnosis of congenital heart defects in the few centers that have such facility. Echocardiograhic findings for CHD in these poor resource, low volume centers, however, are often considered sub-optimal with many false negatives and false positives. This study therefore, was aimed at correlating the preoperative transthoracic echocardiography with the intaoperative findings in patients with congenital heart disease undergoing surgery as well as validating the accuracy and safety of the operative correction of such congenital heart defects using echocardiography alone as a preoperative imaging modality.

## Methods

After institutional clearance, a total of 250 patients who had corrective or palliative surgery for congenital heart disease at the National Cardiothoracic Centre Korle Bu Teaching Hospital, Accra, Ghana, from September 2012 to September 2017, were prospectively followed up during the in-hospital phase. The results of pre-operative trans-thoracic echocardiography were compared with the intra-operative anatomic description as observed by us during surgery. Patients who had invasive preoperative modalities such as cardiac catheterization or CT angiography were excluded from the study.

The preoperative investigation comprised 2-dimensional echocardiography with analysis of intracardiac flows by using pulsed and continuous Doppler and colour-flow mapping. Trans-thoracic echocardiography was performed by paediatric and adult cardiologists with Philip HD 15, SC200 USA and Toshiba TA 510 (Toshiba, Tokyo, Japan), with 2.5, 5.0 and 7.5 MHZ transducers and color flow mapping. In paediatric patients, sedation, when necessary, was performed through the oral or rectal routes using 8% chloral hydrate at doses recommended for the paediatric age group. Evaluation of the thoracic and abdominal situs, determination of the presence of 2 atria and 2 ventricles, superior vena cava, inferior vena cava, pulmonary veins and their respective drainage sides, assessment of the atrial and ventricular septa, morphology and location of the atrioventricular valves, right and left ventricular outflow tracts, arterioventriculalar connections, morphology of the pulmonary artery and proximal branches and morphology of the aortic root were all carried out.

**Fig. 1:**
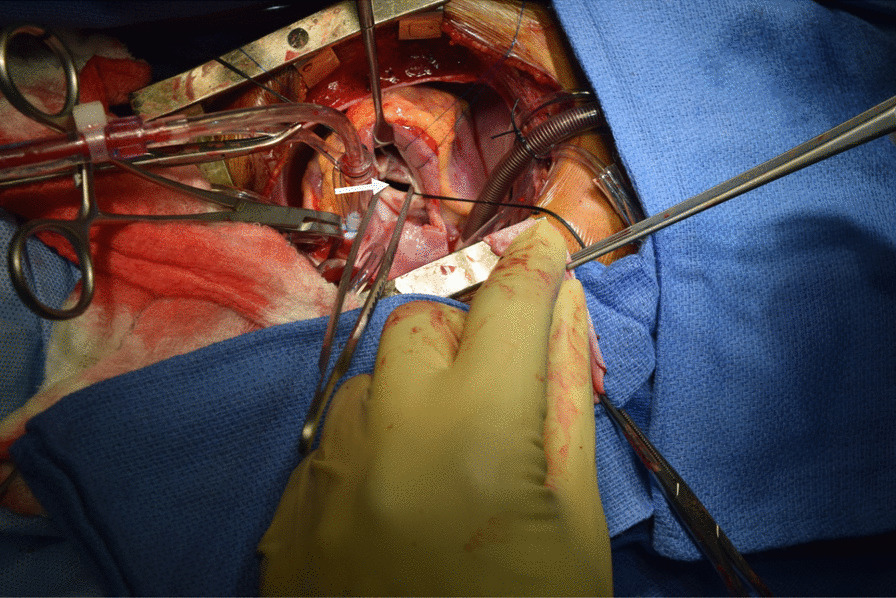
Ventricular septal defect (white arrow)

Operations were performed by a team led by two paediatric and congenital heart surgeons and five adult cardiothoracic surgeons. Surgery was either open heart through median sternotomy and using cardiopulmonary Bypass, with invasive and noninvasive monitoring, aorto- bicaval cannulation, myocardial protection with St.Thomas 2 cardioplegic solution, and autologous pericardial patch repair of lesion (Fig. 1) or closed heart through a thoracotomy for congenital defects such as PDA. The intra-operative anatomic descriptions of the lesions as noted by the attending surgeons were recorded. Post-operative ECHO was done prior to patient’s discharge from hospital to assess correction of defect, residual or missed lesions.

Accuracy of the trans-thoracic ECHO was determined by comparing the data obtained on the pre-operative ECHO and the intra-operative findings. The differences found were divided into false positive (when the ECHO shows a finding not confirmed at surgery) and false negative (when ECHO does not show a feature observed at surgery). The errors were sub-divided into major errors when they led to a significant modification in the surgical procedure or affect the surgical risk or post-operative outcome. Minor errors, were those that did not significantly modify surgical procedure or affect the surgical risk or post-operative outcome.

The analysis included profiling of patients on different demographic and clinical parameters. Quantitative data was presented in terms of means and standard deviation. Student t test was used for comparison of individual quantitative parameters. Cross tables were generated and chi square test was used for testing of associations. *P* value < 0.05 was considered statistically significant. SPSS software was used for analysis.

## Results

**Fig. 2:**
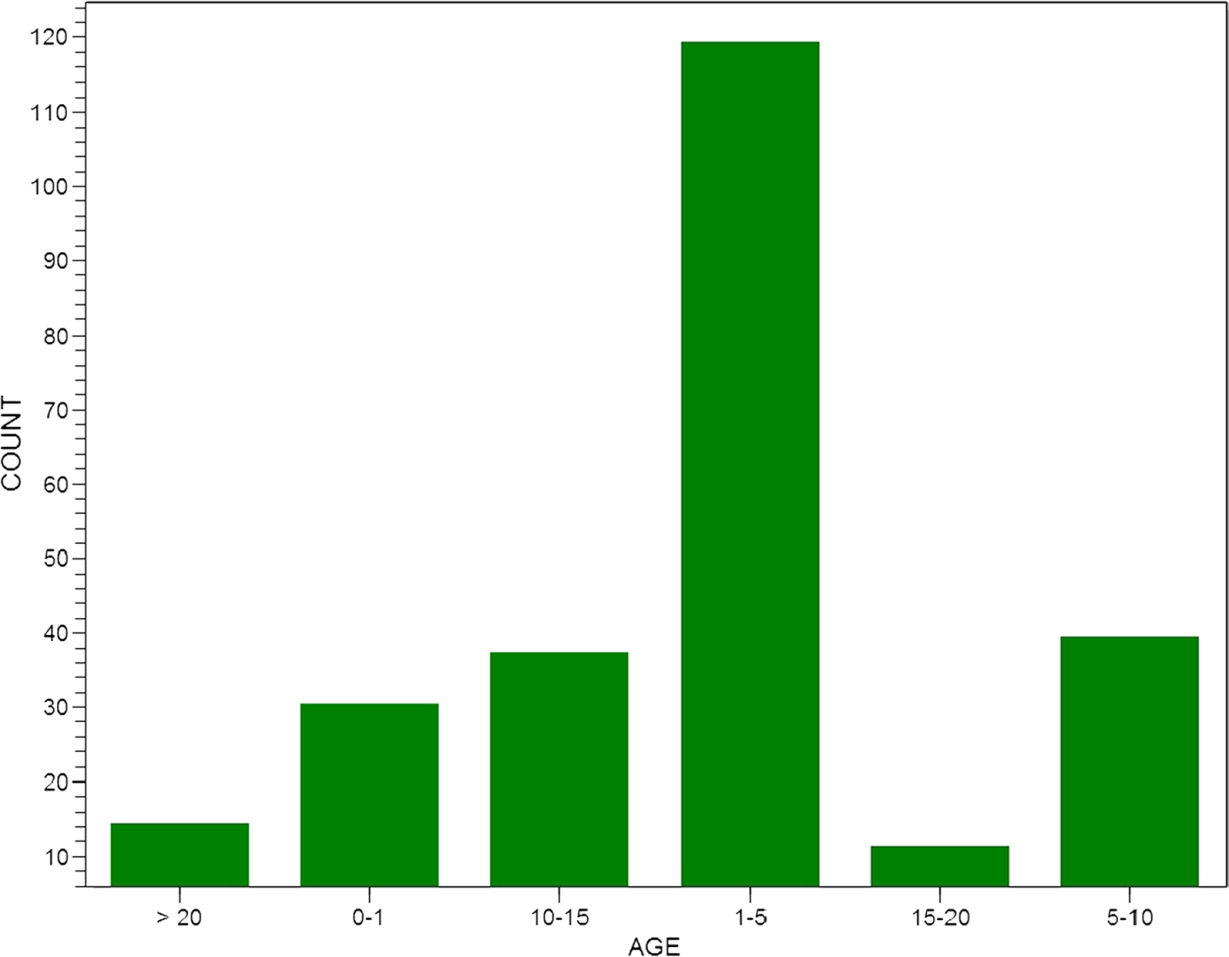
Showing age distribution of patients in years

Of the 250 patients prospectively followed up during the in-hospital phase from September 2012 to September 2017, ages ranged from two (2) months to sixty (60) years (Fig. 2). The mean was 4 years 95 days, median 1 year 180 days. The female sex accounted for 152 (60.6%). The trans-thoracic echocardiography did not show some findings observed at surgery in 19 patients (false negative, 7.6%) as follows: (1) 5 patients had double chambered right ventricle at surgery which echocardiography diagnosed as VSD with pulmonary stenosis and in one patient as TOF. (2) 3 persistent left superior vena cava. (3) 3 Double outlet right ventricle, diagnosed by echocardiography as TOF. (4) 2 aneurysm of sinus of valsalva. (5) 2 supramitral ring. (6) 1 pulmonary stenosis. (7) 1 unroofed coronary sinus. (8) 1 pulmonary atresia. (9) A patient was diagnosed by echocardiography as having bicuspid aortic stenosis and was prepared for aortic valve replacement, but was found to have congenital subvalvular aortic membrane with normal pliable trileaflet aortic valve at surgery. (See Tables 1, 2).

**Table 1:** Distribution of the congenital heart diseases according to the transthoracic echocardiography diagnosis

Lesion (N)	Echo diagnosis isolated lesion (N)			Isolated (N)
	C	F+	F−	
ASD	34	1	3	38
VSD	66	–	9	75
PDA	64	–	–	64
TOF	43	–	6	49
DORV	6	–	–	6
PAVSD	1	–	–	1
ASD + PS	5	2	–	7
VSD + PS	4	–	–	4
ASD + PDA	3	–	–	3

C, Confirmed; F+, False positive( when the echocardiography finding was not confirmed at surgery); F, False negative( when the echocardiogram did not show a finding observed at surgery); ASD, Atrial septal defect; VSD, Ventricular septal defect; PDA, Persistent ductus arteriosus; TOF, Tetralogy of Fallot; DORV, Double outlet right ventricle; PAVSD, Partial atrioventricular septal defect; PS, Pulmonary stenosis

**Table 2:** Distribution of the defects and their respective discrepancies

Lesion	False negative	False positive
ASD	Supramitral ring(2), Unroofed coronary sinus(1)	ASD(1)
VSD	DCRV(4),PL SVC(2),PS	
	Sinus of valsalva aneurysm(2), aortic regurgitation(1)	–
PDA	**–**	**–**
TOF	DORV(3), PA(1),PLSVC(1),DCRV(1)	–
PAVSD	–	–
ASD + PS	–	PS
VSD + PS	–	–
ASD + PDA	–	–

ASD, Atrial septal defect; VSD, Ventricular septal defect; DCRV, Double chambered right ventricle; PLSVC, Persistent left superior vena cava; PS, Pulmonary stenosis; PDA, Persistent ductus arteriosus; TOF, Tetralogy Fallot; DORV, Double out right ventricle; PA, Pulmonary atresia

The echocardiographic findings were not confirmed at surgery in 3 patients (false positive, 1.2%) thus: (1) 2 patients were said to have pulmonary stenosis on echocardiography, which was not seen at surgery. (2) 1 patient was prepared and had surgery for echocardiographic diagnosis of superior sinus venosus ASD which was not found at surgery. The comparison of the false positive results in simple and complex diseases was not statistically significant, but that of the false negative results was statistically significant (*P* < 0.005). Two hundred and thirty seven (95.5%) patients did not have residual lesion on post operative echocardiography, only 4.5% had residual lesion, most of which were small residual VSD and PDA which closed during follow up and non was associated with errors in echocardiography. The trans-thoracic echocardiography diagnosis was completely correct in 228 (91.2%) of patients. Table 2 shows the totally correct results, the false negative and false positive results related to each heart disease. Tables 1 and 2 shows the false negative and false positive results (discrepancies) identified for each type of CHD diagnosed on echocardiography.

## Discussion

In this study, trans-thoracic echocardiography proved to be highly reliable as an exclusively noninvasive imaging modality in the preoperative assessment of patients with CHD undergoing surgery. These findings correlate with those from some cardiac centers where the sensitivity and specificity of echocardiographic diagnosis in the preoperative assessment of patients with CHD is established [1–3, 5, 6, 8], Most studies cited in the literature used both trans-thoracic and trans-oesophageal echocardiography in the preoperative assessment. This study, however, used only trans-thoracic echocardiography as an isolated imaging modality in the preoperative assessment. This is important since only trans-thoracic echocardiography is used in the few centres that have such services in the West African sub-region and this showed from the study to be sufficiently safe as a preoprerative imaging modality to indicate surgery in patients with CHD.

The diagnostic error in identifying extra-cardiac structures such as persistent left superior vena cava in patients with ventricular septal defects and tetralogy of Fallot in this study is thought to be due to the difficulty in assessing the extra-cardiac structures on echocardiography, because such structures have similar echo densities. This was also observed by Lillian et al. [1]. The assessment of extra-cardiac structures through the suprasternal window often used in routine trans-thoracic echocardiography is known to be difficult, mainly in small children. This limitation undoubtedly might have contributed to incomplete diagnosis in patients with persistent left superior vena cava, mainly when no dilatation of the coronary sinus is observed which may suggest anomalous systemic venous drainage, a finding corroborated by Soonswang et al. [7] as well as Lilian et al. [1].

False positive and false negative results were observed in the assessment of patients with CHD with increased blood flow such as VSD and ASD in which double chambered right ventricle and pulmonary valvular stenosis were missed. A finding also reported by Lilian et al. [1, 3–6, 12]. This is thought to be secondary to an over or underestimated assessment of the right ventricular cavity and relative valvular stenosis, in the presence of increased pulmonary blood flow. A more detailed evaluation of the right ventricular cavity and valvular morphology as well as analysis of the subvalvular and postvalvular pulmonary blood flow which will appear with different velocity is suggested.

Assessment of the pulmonary valve may be hindered in patients with TOF with a significant obstruction to right ventricular outflow tract, in which the pulmonary valve is usually hypoplastic as noted in this study in which pulmonary atresia was found at surgery, but missed on echocardiography in a patient with TOF. In that case analysis with pulsed Doppler located right after the pulmonary valve could help to differentiate functional pulmonary atresia from anatomic pulmonary atresia.

Three patients were diagnosed by echocardiography as having TOF, but were found at surgery to have DORV. This is not surprising since both are within the spectrum of CHD with right ventricular out flow tract obstruction, the difference being in the degree of aortic over ride. The literature is however, still controversial about the differentiation between the two. While some authors consider the presence of complete bilateral double infundibulum essential for the diagnosis of DORV, others consider that the criterion hinders the diagnosis of DORV with TOF and would rather choose to define as DORV those cases in which more than 50% of the circumference of the aorta relates to the right ventricle independent of the nature of the structures that support it. Differentiating these two entities is however, important for surgical repair because in TOF, a straight patch may be sufficient for repair of the VSD, while in DORV tunneling of the patch is required. A straight patch in the repair of DORV will result in left ventricular outflow tract obstruction. While it is true that there was a change in operative approach in view of the intra-operative finding, the overall outcome was unaltered in this study.

The false positive result that occurred in a patient with atrial septal defect (ASD), was that of a child whose echocardiography showed that he had an ASD, but at operation no lesion was found. This diagnostic error is an isolated case and the patient is in the category that would benefit from cardiac catheterization or CT angiography, since the absence of a lesion at surgery does not rule out the presence of a lesion. The false negative in relation to ASD included one patient with supra-mitral ring which was not seen on echocardiography but identified at surgery. A patient was also found at surgery to have unroofed coronary sinus which was not diagnosed by echocardiography. The diagnostic impression of atrial septal defect may often occur in adult patients due to the difficulty of the echocardiographic window, because the atrial septum is located very far away from the transducer, resulting in lack of definition of the tissues of the oval foramen which is thicker than the rest of the septum. One patient had autologous VSD pericardial patch dehiscence on post operative day one necessitating reoperation. We noticed pericardial adhesions at initial surgery possibly from previous endocarditis. This was thought to have contributed to this complication.

The possibility of observer error in the results of the echocardiography cannot totally be ruled out considering the fact that echocardiography is operator dependent. It is to be noted however, that none of the errors affected the outcome of surgery significantly. This is a good development for our sub-region where the practice of echocardiography is still evolving.

## Conclusion

Based on the results of this study, preoperative transthoracic echocardiography done by cardiologists at the National Cardiothoracic Center, Korle Bu Teaching Hospital Accra, demonstrated a high correlation with intraoperative findings..

The study also showed that surgery could be performed without previous invasive study even in more complex cases such as tetralogy of Fallot, double outlet right ventricle, AV- canal defects etc. This is because trans-thoracic echocardiography proved to be a highly sensitive, specific and safe diagnostic imaging modality. The drawback however, remains that this investigation is subjective and its reliability also dependent on the competence of the echocardigrapher. In spite of this however, trans-thoracic echocardiography, can be safely used for the preoperative assessment of patients with congenital heart diseases, catheterization being reserved for cases with specific indications.

## Limitations of the study

The echocardiography was done by paediatric and adult cardiologists; bearing in mind that this imaging modality is operator dependent, the margin of error is likely to be higher since it was carried out by more than one operator.

The study was also a single institutional study, a multi-institutional or a regional study would provide more power and any conclusion drown is likely to be a reflection of the true situation.

A comparison of trans-thoracic echocardiography with other modalities such as trans-oesophageal echocardiography or cardiac catheterization would have also provided more facts but these facilities were not available. Cardiac catheterization is only available for adult studies at the in institution.

## Data Availability

Data will not be shared because of institutional policy.

## Abbreviations

CHD: Congenital heart disease
NCTC: National cardiothoracic centre
CT: Computarised tomography
ECHO: Echocardiography
